# Dual-mode operation of 2D material-base hot electron transistors

**DOI:** 10.1038/srep32503

**Published:** 2016-09-01

**Authors:** Yann-Wen Lan, Carlos M. Torres, Jr., Xiaodan Zhu, Hussam Qasem, James R. Adleman, Mitchell B. Lerner, Shin-Hung Tsai, Yumeng Shi, Lain-Jong Li, Wen-Kuan Yeh, Kang L. Wang

**Affiliations:** 1 National Nano Device Laboratories (NDL), Hsinchu 30078, Taiwan; 2 Department of Electrical Engineering, University of California at Los Angeles, Los Angeles, California 90095, United States; 3 Space and Naval Warfare (SPAWAR) Systems Center Pacific, San Diego, California 92152, United States; 4 Nanomedical Diagnostics, Production Division, San Diego, CA 92121, United State; 5 Physical Sciences and Engineering Division, King Abdullah University of Science and Technology (KAUST), Thuwal, 23955-6900, Kingdom of Saudi Arabia; 6 Department of Electrical Engineering, National University of Kaohsiung, Kaohsiung 811, Taiwan; 7 National Nano Device Laboratories (NDL), National Applied Research Laboratories, Taipei 10622, Taiwan

## Abstract

Vertical hot electron transistors incorporating atomically-thin 2D materials, such as graphene or MoS_2_, in the base region have been proposed and demonstrated in the development of electronic and optoelectronic applications. To the best of our knowledge, all previous 2D material-base hot electron transistors only considered applying a positive collector-base potential (V_CB_ > 0) as is necessary for the typical unipolar hot-electron transistor behavior. Here we demonstrate a novel functionality, specifically a dual-mode operation, in our 2D material-base hot electron transistors (e.g. with either graphene or MoS_2_ in the base region) with the application of a negative collector-base potential (V_CB_ < 0). That is, our 2D material-base hot electron transistors can operate in either a hot-electron or a reverse-current dominating mode depending upon the particular polarity of V_CB_. Furthermore, these devices operate at room temperature and their current gains can be dynamically tuned by varying V_CB_. We anticipate our multi-functional dual-mode transistors will pave the way towards the realization of novel flexible 2D material-based high-density and low-energy hot-carrier electronic applications.

Since 1960, ballistic hot electron transistors (HETs) have been vigorously researched and implemented in diverse material systems (e.g. cold cathode transistor exploiting a thin metal base^1^,^2^, planar doped barrier transistor incorporating III-V compound semiconductors^3^, two-dimensional electron gas (2DEG)-based HETs^4^,^5^,^6^, etc.) for their potential in high-speed applications. Analogous in design to a bipolar transistor, HETs are comprised of an emitter, base, and collector. However, various properties of the injected ballistic hot electrons, such as their initial velocity, higher kinetic energy, and quasi-mono-energetic distribution upon injection via quantum tunneling, differ from the diffusive transport in bipolar transistors^2^,^7^. In HETs, the ballistic hot electrons are injected through a thin tunnel barrier separating the emitter from the base, and a portion of these hot electrons are collected upon traversing a filter barrier at the base-collector junction (e.g. contribute towards the on-state collector current).

Furthermore, the cutoff frequency of HETs is primarily governed by the base thickness and the resistances and capacitances of the emitter and collector regions. To this end, various bulk semiconductor heterostructures, such as InGaAs/InP and AlGaAs/GaAs, have been precisely engineered with undoped and narrow (<100 nm) base regions since the 1970 s with the introduction of advanced epitaxial technologies, such as molecular beam epitaxy (MBE) and metal-organic chemical vapor deposition (MOCVD)^7^. However, several issues including inelastic electron scattering in the finite-width base region, finite base transit time, and quantum-mechanical reflections (e.g. impedance-mismatching) at the collector-base junction typically resulted in subpar current gains at or below room temperature^2^,^4^,^5^. In addition, these epitaxial techniques add to the complexity in the time and cost of fabricating such structures.

The advent of 2D van der Waals materials^6^, such as graphene^7^ and the transition metal dichalcogenides^8^,^9^,^10^,^11^,^12^,^13^ (TMDs), has sparked a paradigm shift in the design and engineering of atomic-scale systems. Their strong in-plane mechanical stability in addition to their weak out-of-plane van der Waals forces allow us to amalgamate atomic-scale heterostructures^11^ exhibiting novel optoelectronic phenomena^14^,^15^ and functionalities^16^,^17^,^18^,^19^. Recently, ballistic hot electron transistors incorporating either monolayer graphene or monolayer MoS_2_ in the base region have achieved high current modulation^20^,^21^ (I_ON_/I_OFF_ ~ 10^4^–10^5^) and high-current gain^22^ (α ~ 0.95) at room temperature, respectively. This unique class of 2D material-base hot electron transistors (2D-HETs) shows great potential for 2D material-based high-frequency logic applications upon further device optimization^16^,^23^,^24^,^25^,^26^.

The 2D-HETs rely upon the vertical (e.g. out-of-plane) emission of hot electrons through an atomic-scale base region and the subsequent filtering of these hot electrons by a built-in potential energy barrier near the base-collector junction. However, in spite of these accomplishments, there is a dearth of insight into the actual out-of-plane transport (e.g. the dominant scattering mechanisms and the actual potential energy landscape) experienced by the hot electrons in these 2D-HETs^27^,^28^,^29^. As far as we know, all previous 2D-HETs operated under the application of a positive collector-base potential (V_CB_ > 0) and thus were limited to a single functionality, namely the typical unipolar hot electron transistor behavior^20^,^21^. To augment the functionality of electronics such as multi-level cells for low footprint vertical transport-based memory applications, here we introduce an alternative and peculiar conduction mode of operation which we refer to as a dual-mode operation in our 2D-HETs upon application of either a positive collector-base potential (V_CB_ > 0) or a negative collector-base potential (V_CB_ < 0). Thus, our 2D-HETs can operate in either a hot-electron or a reverse-current dominating mode depending upon the particular bias configuration. The 2D-HETs operate at room temperature and their current gains can be dynamically tuned by varying V_CB_. Furthermore, we surmise that the current saturation-like behavior in the transfer characteristics of the MoS_2_-HETs when operated in the reverse-current dominating mode (V_CB_ < 0) could serve as a multi-level cell (e.g. data storage) in future multi-functional 2D material-based high-density and low-energy hot-carrier electronic (e.g. vertical transport based logic and memory) applications.

## Results

**Figure 1:**
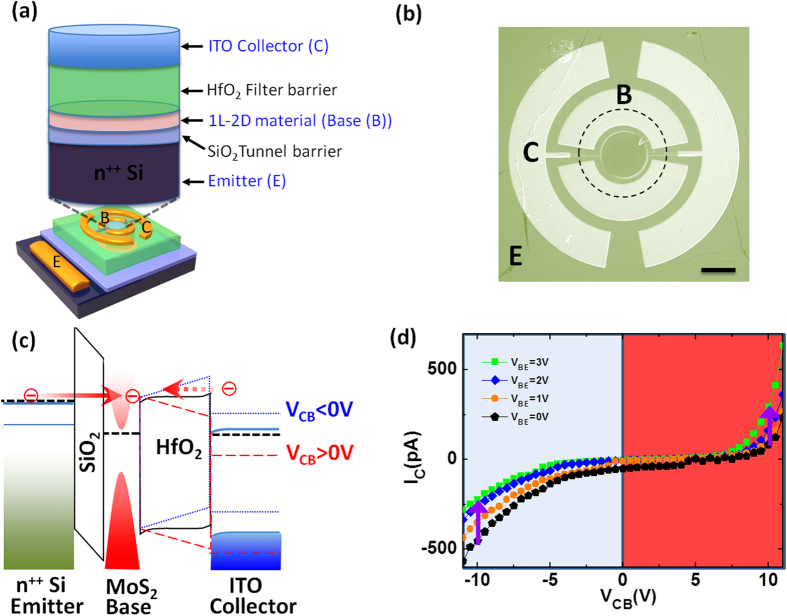
Device structure and energy band diagram of the MoS_2_-HET. (**a**) An isometric view of an MoS_2_-HET device structure. The capital letters E, B, and C represent the emitter, base, and collector, respectively. (**b**) Optical micrograph (top-view) of an actual MoS_2_-HET device. The scale bar is 100 μm. The dashed circle outlines the MoS_2_ region. (**c**) Energy band diagram depicting the collector current contributions at the on-state condition for two different polarities of the collector-base voltage. For V_CB_ > 0 (dashed red lines), the hot-electrons tunneling through the emitter-base tunnel barrier have sufficient kinetic energy to overcome the filter barrier and reach the collector, whereas for V_CB_ < 0 (dotted blue lines), electrons flow from the ITO to the base region to form a reverse base-collector current. (d) Common-base output characteristics. The collector current is shown as a function of V_CB_ at V_BE_ = 0 V, +1 V, +2 V, and +3 V.

We demonstrate vertical transport 2D-HETs which exhibit a novel dual-mode operation by incorporating either monolayer MoS_2_ (MoS_2_-HET) or monolayer graphene (G-HET) in the base region. The device structure of the 2D-HET is presented in Fig. 1a and a top-view optical micrograph of an actual MoS_2_-HET is shown in Fig. 1b. The three-terminal device consists of a degenerately-doped n^++^ silicon substrate (N_D_ ~ 10^19^ cm^−3^) as the emitter (E), a monolayer of chemical vapor deposition (CVD) grown 2D material (e.g. either MoS_2_ or graphene) as the base (B), and sputtered (~45 nm) ITO as the collector (C). A thermally grown thin (~3 nm) SiO_2_ tunnel barrier separates the emitter and base terminals, whereas an atomic-layer deposited (~55 nm) HfO_2_ separates the base and collector and serves as the filtering barrier. The detailed fabrication process is described in the Methods and in our previous work^22^. In this particular study, a common-base configuration was employed during the electrical measurements. Note that both of the base contacts are grounded during the electrical measurements in order to achieve a uniform potential distribution across the MoS_2_ base region.

We first focus on describing the two modes of operation for the 2D-HETs using energy band diagrams in order to clearly understand the physics governing the device transport. The 2D-HET with monolayer MoS_2_ as the base (MoS_2_-HET) will serve as an example. Figure 1c shows the energy band diagram for the off-state and the on-state conditions of the MoS_2_-HETs. In the absence of an applied V_CB_, most of the hot-electrons injected through the tunnel oxide have insufficient kinetic energy to overcome the filter barrier at the collector-base junction and do not reach the collector. Instead, they back-scatter and thermalize into the MoS_2_ base region. However, the situation drastically changes with the application of a large V_CB_. There are two possible cases for the on-state condition of the MoS_2_-HETs, depending upon the polarity of the applied V_CB_. The first case describes the typical hot-electron injection behavior and occurs for V_CB_ > 0. In this scenario, hot-electrons tunneling through the emitter-base tunnel oxide have sufficient kinetic energy to overcome the filter barrier, reach the collector, and contribute to the collector current (I_C_). The second case describes a reverse-current behavior, which is a novel feature and mode of operation enabled by our 2D-HETs, and occurs for V_CB_ < 0. In this scenario, the injected hot-electrons tunneling from the emitter do not have sufficient kinetic energy to surpass the raised filter barrier. Subsequently, these electrons are back-scattered and accumulate within the 2D material-base region which serves to suppress the base-collector reverse-current (I_C_). Interestingly, ΔI_C_, which denotes the amount of change in the base-collector current due to the hot electron injection from the emitter, can be tuned with the applied V_BE_ in this mode of operation. Figure 1d shows the common-base output characteristics of one of our MoS_2_-HETs. The collector current (I_C_) is shown as a function of V_CB_ at various V_BE_. It is evident that I_C_ increases at a large positive V_CB_ whereas I_C_ is suppressed at a large negative V_CB_. Thus, by adjusting the polarity of V_CB_, it is possible to operate the 2D-HETs such that their collector current is mainly contributed by either hot-electrons originating from the emitter or electrons originating from the collector. Since ΔI_C_ is the change in the collector current caused by the injection of the hot electron input current (e.g. an increasing magnitude of V_BE_ > 0 modulates ΔI_C_), it will be used instead of I_C_ for the discussion of the dual-mode operation for the remainder of this report.

### Hot-electron dominating mode of operation in the MoS_2_-HETs

**Figure 2:**
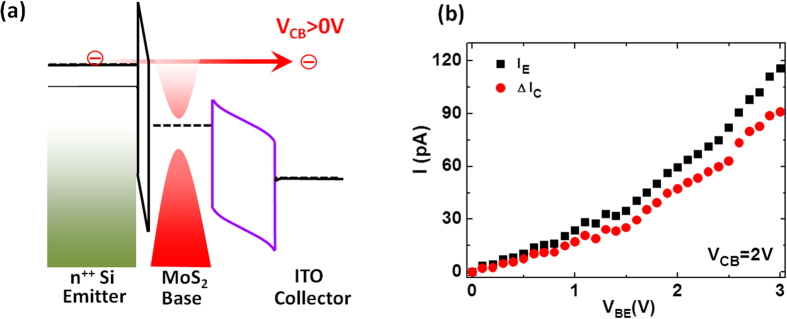
MoS_2_-HET operating in the hot-electron dominating mode. (**a**) Energy band diagram depicting the MoS_2_-HETs operating in the hot-electron dominating mode. The conduction and valence band edges at the collector-base junction are shown for a positive V_CB_, which reduces the filter barrier for the hot-electrons. (**b**) Input and transfer characteristics for an MoS_2_-HET. The emitter current (black squares) and the collector current (red circles) are shown as a function of V_BE_ at V_CB_ = +2 V.

We first characterize the MoS_2_-HET in the hot-electron dominating mode of operation by applying positive V_CB_. Figure 2a shows the energy band diagram depicting the conduction and valence band edges at the collector-base junction with a positive V_CB_ applied. In this mode of operation, once hot-electrons tunneling through the emitter-base tunnel barrier have sufficient kinetic energy, they can vertically transport through the MoS_2_ base region, surpass the filter barrier at the collector-base junction, and reach the collector. Consequently, an increasingly positive V_CB_ will continue to effectively make the filter potential barrier thinner and promote hot-electrons reaching the collector due to an increase in their transmission probability. This qualitative behavior is exhibited in the input and transfer characteristics of the MoS_2_-HETs. The input characteristics (I_E_-V_BE_) correspond to how the emitter current depends on V_BE_, whereas the transfer characteristics (I_C_-V_BE_) correspond to the manner in which the collector current varies with V_BE_. Figure 2b shows the input and transfer characteristics for one of MoS_2_-HETs. The emitter current (I_E_) and the collector current (I_C_) are shown as a function of V_BE_ (V_BE_ was swept from 0 to +3 V) at a V_CB_ of +2 V. Both currents rapidly increase at larger V_BE_, as is typical for HETs. From the input and transfer characteristics, the common-base current gain (α) of this device can be determined, which is a figure of merit for HETs and is defined as α = I_C_/I_E_. For this particular device and biasing condition of V_CB_ = +2 V and V_BE_ = +3 V, α is about 0.81, which implies that at least 80% of the injected hot-electrons ballistically traverse the single-layer MoS_2_ base region at room temperature. Further details concerning the hot-electron dominating mode of operation in the MoS_2_-HETs is mentioned in our previous work^22^.

### Reverse-current mode of operation in the MoS_2_-HETs

**Figure 3:**
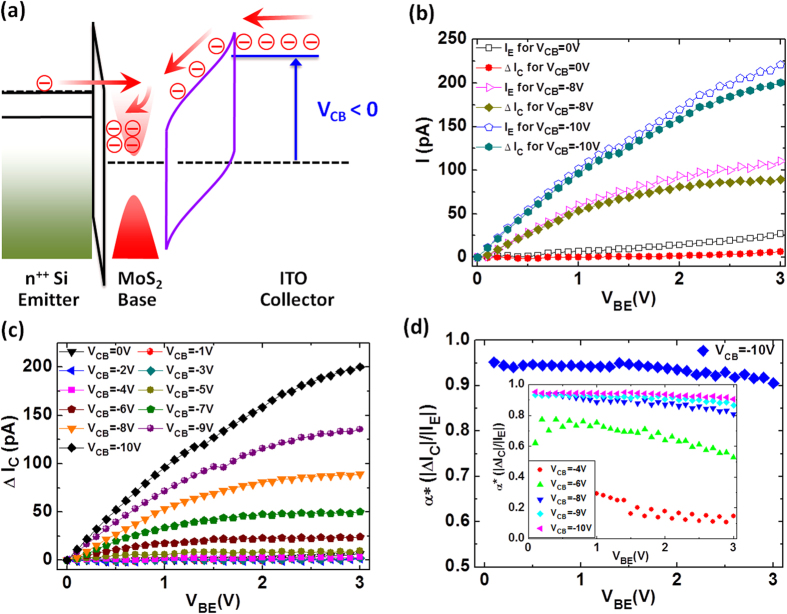
MoS_2_-HET operating in the reverse-current mode. (**a**) Energy band diagram depicting the MoS_2_-HETs operating in the reverse-current mode. Electrons flow from the degenerately n-doped ITO conduction band to the MoS_2_ base region upon application of a reverse-bias across the collector-base junction (V_CB_ < 0). The conduction and valence band edges at the collector-base junction are shown for a negative V_CB_, which raises the filter barrier experienced by the hot-electrons tunneling from the emitter and subsequently promotes an electron build up in the base region. (**b**) Input and transfer characteristics for an MoS_2_-HET operating in the reverse-current mode. The emitter current (open symbols) and the collector current (filled symbols) are shown as a function of V_BE_ at V_CB_ = 0, −8 and −10 V. (**c**) Transfer characteristics for an MoS_2_-HET. The reverse base-collector current as a function of V_BE_ is shown for V_CB_ from 0 to −10 V with step of −1 V. (**d**) α* as a function of V_BE_ at V_CB_ = −10 V. The inset shows α* as a function of V_BE_ at V_CB_ = −4, −6, −8, −9, and −10 V.

Shifting from the hot-electron dominating mode of the MoS_2_-HET, we next investigate the device characteristics operating under the reverse-current dominating condition. Figure 3a shows the energy band diagram depicting the reverse-current mode of operation for the MoS_2_-HET. Specifically, Fig. 3a shows the conduction and valence band edges at the collector-base junction with a negative V_CB_ applied. In this mode of operation, the increasingly negative V_CB_ drives more and more electrons to flow from the degenerately n-doped ITO conduction band, past the filter barrier and into the base region, thus forming the reverse-current. With the injection of hot-electrons from the emitter, the continuously increasing filter barrier (V_CB_ < 0) causes these hot-electrons to have insufficient kinetic energy to reach the collector and thus they back-scatter into the MoS_2_ base region. Consequently, these back-scattered electrons build up in the MoS_2_ base region which cause a deficiency in the available density of states in the MoS_2_ and thereby decrease or suppress the reverse-current flowing into the base region from the degenerately n-doped ITO conduction band. The change or modulation in the reverse base-collector current (I_C_) caused by the injected hot-electrons from the emitter into the MoS_2_ base region is denoted as ΔI_C_. This reverse base-collector current (ΔI_C_) can be modulated with V_BE_ by tuning the amount of hot-electrons that are injected from the emitter and which eventually build up in the MoS_2_ base region. In the reverse-current mode of operation (V_CB_ < 0), we define the effective current gain: α* = |Δ*I*_*C*_|/|*I*_*E*_| as the ratio of the measured reverse base-collector current to the injected hot-electron emitter current in the MoS_2_ base region, where ΔI_C_ is defined as the suppressed reverse base-collector current arising from the build-up of the injected hot-electrons in the MoS_2_ base region. Thus, our MoS_2_-HETs, when biased in the reverse-current dominating mode, enable the dynamic control of the available density of states in the 2D base region by varying V_BE_. This qualitative behavior for the reverse base-collector current mode of operation is exhibited in the input and transfer characteristics of the MoS_2_-HETs. Figure 3b shows the input and transfer characteristics for one of the MoS_2_-HETs biased at three different V_CB_ (V_CB_ = 0, −8, and −10 V). It is evident that both the emitter and collector currents increase with larger negative V_CB_. Similarly, Fig. 3c shows a family of transfer characteristics, with the suppressed reverse base-collector current (ΔI_C_) as a function of V_BE_ shown for various negative V_CB_. The transfer characteristics of the MoS_2_-HET when operated in the reverse-current mode (V_CB_ < 0) are peculiar in that the reverse base-collector current tends to saturate with increasing V_BE_. Additionally, the reverse-current magnitude increases with larger negative V_CB_ bias. We speculate that this novel current saturation-like behavior could serve as a multi-level cell for low footprint vertical transport-based memory^30^,^31^,^32^,^33^,^34^,^35^applications in the future. As an example, consider biasing the 2D-HET at V_BE_ = +3 V (e.g. the highest hot electron injection current to avoid dielectric breakdown of the tunnel barrier). We can vary the steady-state reverse-current (ΔI_C_) by setting V_CB_ < 0 to various values. Based on Fig. 3c, we can address distinguishable (ΔI_C_) charge states for V_CB_ from −6 V to −10 V and thus encode at least 4 states for a minimum of a 2-bit memory cell. Multi-level cells are memory units capable of storing more than one bit of information and thus can result in lower cost per unit of storage and higher data storage density. Furthermore, it was recently shown that cheaper multi-level cell flash drives used in practice are just as reliable as more expensive single-level cells^36^. Thus, our dual-mode 2D-HETs may find opportunities as ultra-dense multi-functional logic/memory units. From the input and transfer characteristics, we can next ascertain the effective current gain (α*) of this device for the reverse-current dominating mode of operation. Figure 3d shows α* for this MoS_2_-HET as a function of V_BE_ at V_CB_ = −10 V. Such a large negative V_CB_ significantly raises the filter barrier height for the injected hot-electrons originating from the emitter, which causes them to back-scatter into the MoS_2_ base region and build up, leading to the effective suppression of the reverse base-collector current. Similar to the hot-electron dominating mode of operation in our previous paper^22^, it is evident that α* exhibits a nearly constant characteristic at all V_BE_ with a value of at least 90% for this particular MoS_2_-HET biased at V_CB_ = −10 V. The inset of Fig. 3d shows a family of α* characteristics as a function of V_BE_ at several negative V_CB_ (V_CB_ = −4, −6, −8, −9, and −10 V). The effective current gain, α*, increases with negative V_CB_ and exhibits a nearly constant characteristic throughout the entire V_BE_ range with a magnitude of about 94% at V_CB_ = −10 V.

### Output characteristics and tunable current gain in the MoS_2_-HETs

**Figure 4:**
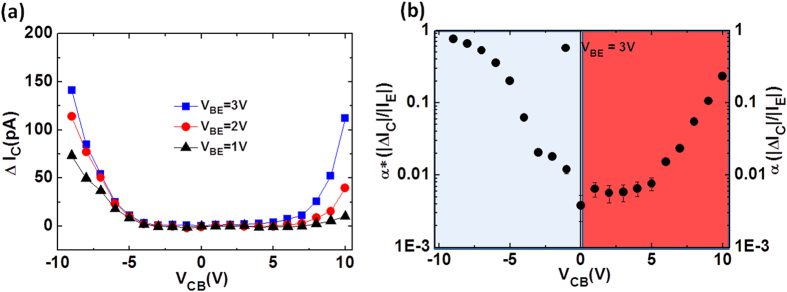
Output characteristics and tunable current gain of the MoS_2_-HET. (**a**) Common-base output characteristics for an MoS_2_-HET. The collector current is shown as a function of V_CB_ at V_BE_ = +1 V, +2 V, and +3 V. (**b**) Both the common-base current gain (α) and the effective current gain (α*) for an MoS_2_-HET are shown in log-scale as a function of V_CB_ for V_BE_ = +3 V.

With the analysis of the input and transfer characteristics complete, we now investigate the common-base output characteristics of the MoS_2_-HETs, which correspond to how the output collector current depends on V_CB_. In order to clearly present the dual-mode operation of our MoS_2_-HETs, the base-collector leakage current when V_BE_ = 0 was subtracted from the measured collector current. Figure 4a shows the common-base output characteristics for one of the MoS_2_-HETs. The collector current is shown as a function of V_CB_ at three positive V_BE_ biases. The dual-mode operation is evident as the device is biased in either the hot-electron (V_CB_ > 0) or the reverse-current (V_CB_ < 0) dominating mode of operation. Above a critical electric field across the HfO_2_, the collector current is quite sensitive to modulation and rapidly increases with a further increase in V_CB_ for both cases of V_CB_ > 0 and V_CB_ < 0. Based on Fig. 4a, the on-off current ratio (I_ON_/I_OFF_) is about 140 when V_CB_ = −10 V and V_BE_ = +3 V, whereas I_ON_/I_OFF_ ~ 125 when V_CB_ = +10 V and V_BE_ = +3 V. In order to convey the robust and dual-mode operation of our MoS_2_-HETs, Fig. 4b shows a semi-log plot of the current gain as a function of V_CB_ at positive V_BE_ = +3 V, which is biased in both the hot-electron (α; V_CB_ > 0) and the reverse-current (α*; V_CB_ < 0) dominating modes of operation. The effective current gain, α*, increases with larger negative V_CB_ as a result of a suppression in the reverse-current and reaches a very high-current gain with a value of about 90% at V_CB_ = −9 V. It is evident that α* can be tuned around two orders of magnitude by varying V_CB_. A similar dependence of α on V_CB_ for the hot-electron dominating case exists as shown in the right portion of Fig. 4b. In the hot-electron dominating mode of operation (V_CB_ > 0), α increases with an increasingly positive V_CB_ and can be tuned over an order of magnitude since this lowers the filter potential barrier experienced by the hot-electrons and allows them to reach the collector.

### Output characteristics and tunable current gain in the graphene-HETs

**Figure 5:**
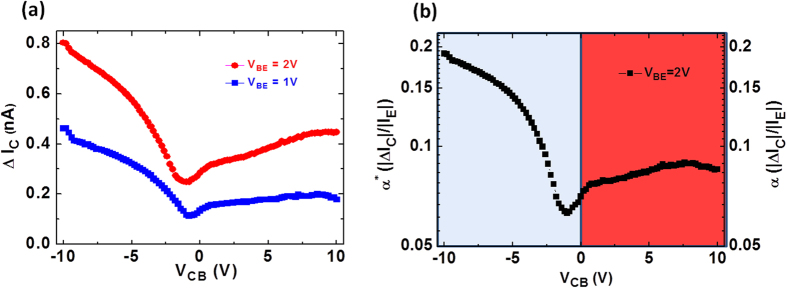
Output characteristics and tunable current gain of the G-HET. (**a**) Common-base output characteristics for a G-HET. The collector current is shown as a function of V_CB_ at V_BE_ = +1 V and +2 V. (**b**) Both the common-base current gain (α) and the effective current gain (α*) for a G-HET are shown in log-scale as a function of V_CB_ at V_BE_ = +2 V.

Furthermore, in order to demonstrate the novel dual-mode operation enabled by our 2D material-base hot electron transistors, we shall now investigate our G-HETs. Figure 5a shows the common-base output characteristics in one of our G-HETs, which is biased in both modes of operation. The collector current is shown as a function of V_CB_ at two positive V_BE_ biases. It is evident that a dual-mode operation is also observed in the G-HETs as the devices are biased in either the hot-electron (V_CB_ > 0) or the reverse-current (V_CB_ < 0) dominating modes of operation. Based on Fig. 5a, the on-off current ratio (I_ON_/I_OFF_) is about 3 when V_CB_ = −10 V and V_BE_ = +2 V, whereas I_ON_/I_OFF_ ~ 2 when V_CB_ = +10 V and V_BE_ = +2 V. The current gain (α) increases with larger positive V_CB_ as a result of a reduction of the filter barrier in the hot-electron dominating process, whereas the effective current gain (α*) increases with larger negative V_CB_ due to suppression in the reverse base-collector current. Additionally, we observed that the current gain can be tuned by varying V_CB_ as shown in Fig. 5b. Hence, by biasing either the MoS_2_-HETs or G-HETs in the common-base configurations, we have explicitly shown the existence of a dual-mode operation and a tunable current gain in our new class of 2D material-base hot electron transistors. Nevertheless, the profiles of both the collector current and the current gain in the output characteristics of the MoS_2_-HET and the G-HET are quite different. At this time, not much is known of the actual out-of-plane transport (e.g. the dominant scattering mechanisms and the actual potential energy landscape) experienced by the hot electrons in these 2D-HETs^27^,^28^,^29^. What we do know is that these are two very different materials (e.g. feature different conduction band offsets, etc.). Monolayer graphene lacks a bandgap and features a linear dispersion relation, whereas monolayer MoS_2_ has a direct bandgap and features a parabolic dispersion relation at the K and K’ points in the Brillouin zone. Furthermore, the effective mass of the electrons travelling perpendicular to the graphene was predicted to be ~ 25–30 m_o_ in a seminal paper^37^. A few reasons for the particularly low current gain in the G-HET compared to the MoS_2_-HET, may be due to the fact that the graphene-HfO_2_ interface features a much higher filter barrier height (e.g. 2.05 eV) compared to that of the MoS_2_-HfO_2_ interface (e.g. 1.52 eV) as well as the possibility of more prevalent acoustic phonon scattering near the base-collector junction for graphene than for MoS_2_. Clearly, further investigations into the out-of-plane transport among different 2D materials and their contact with bulk dielectrics will greatly benefit future device optimization. In the mean time, further improvement in the device performance of the 2D-HETs will be directed towards increasing the injected tunneling current density to a more suitable level for practical applications. This can be achieved via fine tuning of the thickness, barrier height, and uniformity of the tunnel barrier, implementation of bilayer insulator tunnel barrier^38^, as well as lowering the contact resistance between the 2D material and the metallic contact leads (e.g. via chemical doping^39^ or 1D edge contact to 2D materials^40^).

## Summary

In conclusion, we have demonstrated a novel vertical dual-mode 2D material-base hot-electron transistor (2D-HET) incorporating either monolayer MoS_2_ (MoS_2_-HET) or monolayer graphene (G-HET) in the base region. This new class of 2D-HETs can operate in either a hot-electron or a reverse-current dominating mode depending upon the particular bias configuration. For the hot-electron dominating mode of operation (V_CB_ > 0), once the hot-electrons tunneling through the emitter-base tunnel barrier have sufficient kinetic energy, they can vertically transport through the 2D material base region, surpass the filter barrier at the collector-base junction, and reach the collector. For the reverse base-collector current dominating mode of operation (V_CB_ < 0), the continuously increasing filter barrier precludes the injected hot-electrons from having sufficient kinetic energy to reach the collector, hence they back-scatter into the 2D material base region. Consequently, these back-scattered electrons build up in the 2D material base region, which induces a deficiency in the available density of states in the 2D material, thereby reducing the reverse base-collector current. Furthermore, these 2D-HETs operate at room temperature and their current gains can be dynamically tuned by varying V_CB_. This dual functionality is enabled by incorporating 2D materials in the base region of the HET structure and by varying the polarity of V_CB_. We anticipate our transistors will pave the way towards the realization of novel flexible 2D material-based high-density and low-energy hot-carrier electronic applications.

## Methods

In this work, we commenced the fabrication process with a 100 mm degenerately-doped n^++^ (N_D_ ~ 1 × 10^19^ cm^−3^) silicon wafer and performed a standard LOCal Oxidation of Silicon (LOCOS) procedure in order to define arrays of active areas for the 2D material-HETs, which were isolated from each other by a 300 nm thick SiO_2_ field oxide. With the silicon surface of the active areas exposed, we then thermally grew a thin ~3 nm SiO_2_ tunnel oxide. Afterwards, we transferred either a large-area CVD monolayer MoS_2_ or graphene on top of the substrate (e.g. SiO_2_ field oxide) so that the particular 2D material covered several arrays of active areas of the 2D material-HETs. The large-area monolayers of MoS_2_ and graphene were grown using CVD methods^41^,^42^ and transferred onto the substrates using PMMA transfer methods^43^,^44^. Subsequently, a photolithography step was performed to mask circular regions of the 2D material covering the active areas. The 2D material outside of the active regions was etched in order to isolate the various devices. The 2D material area of each device is about 8 × 10^4^ μm^2^. A second photolithography step was performed in order to pattern and deposit the base contacts (20 nm thick Ti/100 nm thick Au for MoS_2_-HETs or 20 nm thick Cr/100 nm thick Au for G-HETs). A 1 nm thick Ti seed layer was evaporated on top of the 2D material and naturally oxidized in air, followed by atomic layer deposition (ALD) of a 55 nm thick HfO_2_ as the filtering barrier. A third photolithography step was performed in order to define a central circular top-gate (e.g. collector) region which encompasses the entire active area of the 2D material-HETs. We then RF sputtered 45 nm of ITO at room temperature into this circular region followed by lift-off. Finally, a fourth photolithography step was performed in order to pattern and deposit the side electrodes (150 nm thick Al/50 nm thick Au) on top of the filtering barrier dielectric. These metallic side electrodes intimately contact the central ITO collector region and allow for easy probing and biasing of the 2D material-HETs. Electrical measurements were performed with a Keithley 4200 Semiconductor Characterization System. All measurements were performed in air and at 300 K. The leakage current was subtracted for all of the data presented in the main text. Specifically, the base-collector leakage current when I_E_ = 0 was subtracted from the measured collector current when biased in the common-base configuration.

## Additional Information

**How to cite this article**: Lan, Y.-W. *et al*. Dual-mode operation of 2D material-base hot electron transistors. *Sci. Rep.* **6**, 32503; doi: 10.1038/srep32503 (2016).
